# Early Plasma Exchange for Multiple Organ Failure Following Massive Wasp Stings: A Case Report

**DOI:** 10.7759/cureus.105595

**Published:** 2026-03-21

**Authors:** Kaichi Kawai, Yoshinori Matsuoka, Kento Izuta, Ryutaro Seo, Koichi Ariyoshi

**Affiliations:** 1 Department of Emergency Medicine, Kobe City Medical Center General Hospital, Kobe, JPN

**Keywords:** envenomation, multiple organ failure, plasma exchange, renal replacement therapy, rhabdomyolysis, wasp stings, wasp venom

## Abstract

Most wasp stings cause only mild local symptoms; however, in rare cases, venom-induced multiple organ failure (MOF) leads to life-threatening systemic complications for which no standardized treatments exist. Wasp venom contains enzymes, amines, and peptides that cause rhabdomyolysis, acute kidney injury (AKI), hepatotoxicity, and disseminated intravascular coagulation (DIC). Extracorporeal blood purification therapies, including therapeutic plasma exchange (TPE), have been proposed as potential treatments, although their indications and efficacy remain unclear.

In this report, we present a case of severe MOF following multiple hornet stings that was successfully treated with early TPE, with the aim of highlighting the role of early TPE in patient survival. A 78-year-old woman was stung by a swarm of hornets during a mountain hike and became immobilized. Emergency medical services found her in shock with impalpable radial pulses and transported her to our hospital. On arrival, her vitals were as follows: Glasgow Coma Scale (GCS) score, E3V4M6; heart rate, 82 beats/min; blood pressure, 84/51 mmHg; respiratory rate, 28 breaths/min; and oxygen saturation, 100% while receiving 10 L/min of oxygen via a reservoir mask. A total of 78 sting sites were identified, with 11 retained stingers on the scalp. Initial laboratory findings revealed hepatic injury (aspartate aminotransferase (AST), 2,236 U/L; alanine aminotransferase (ALT), 628 U/L; and lactate dehydrogenase (LDH), 1,143 U/L), renal dysfunction (blood urea nitrogen (BUN), 23.8 mg/dL and creatinine (Cr), 1.14 mg/dL), coagulopathy (prothrombin time-international normalized ratio, 1.64 and activated partial thromboplastin time >200 s), and gross hematuria. Diagnosed with anaphylactic shock, she was immediately treated with intramuscular adrenaline (0.3 mg), corticosteroids, and antihistamines. All retained stingers were promptly removed. Approximately four hours after admission, her hepatic and renal functions deteriorated further. A single session of TPE was performed eight hours after admission, during which approximately 2.4 L of plasma was replaced with 20 units of fresh frozen plasma. After TPE, hepatic and renal functions gradually improved. Oxygen demand transiently increased, requiring a non-rebreather mask at 9 L/min; however, intubation was avoided. On day 2, liver enzyme levels peaked (AST, 2,362 U/L; ALT, 742 U/L), renal function improved, and creatine kinase (CK) levels rose to 19,815 U/L. On day 4, CK levels peaked at 39,239 U/L and subsequently declined. The patient was transferred from the intensive care unit to a general hospital ward on day 5 and was discharged on day 22 following full recovery.

In severe hornet envenomation with systemic complications, such as MOF and DIC, early intensive care with TPE may be beneficial. Further studies are required to clarify the optimal timing, modality, and indications for blood purification therapies in wasp venom toxicity.

## Introduction

In clinical practice, wasp stings are frequently encountered, and most cases present with mild localized symptoms, such as pain and swelling. However, some cases develop systemic involvement and severe complications [1]. Although anaphylactic shock is the most widely recognized mechanism of severe systemic reactions, venom-induced multiple organ failure (MOF) has also been acknowledged as a serious clinical condition [2]. Wasp venom, including that from hornets (*Vespa* spp.), comprises a complex mixture of biologically active components, including enzymes (e.g., phospholipases and hyaluronidase), peptides, and biogenic amines. These components can induce systemic toxicity through direct cytotoxic effects and inflammatory responses, leading to complications such as rhabdomyolysis, acute kidney injury (AKI), hepatotoxicity, coagulopathy, and, in severe cases, MOF [3-5]. AKI is one of the most common systemic complications of wasp stings, with an estimated incidence of approximately 21% [6]. Furthermore, the mortality rate among patients sustaining more than 10 stings has been reported to be 5.2%, and the overall mortality associated with progressive systemic manifestations of wasp venom toxicity has been reported to reach up to 44% [7].

In severe cases, intensive care is warranted; however, to our knowledge, no standardized treatment protocol currently exists. Among promising therapeutic options, extracorporeal blood purification therapies have attracted attention owing to their potential to remove circulating toxins and mitigate the progression of MOF. Plasma exchange, in particular, is capable of removing high-molecular-weight venom components, myotoxins, and proinflammatory cytokines that contribute to organ injury - components that conventional renal replacement therapies are generally unable to eliminate. This makes plasma exchange a theoretically appealing option in the acute phase of envenomation.

In this report, we present a case of rapidly progressive MOF following massive envenomation by hornet stings in which early therapeutic plasma exchange (TPE) contributed to the patient’s survival. While previous reports often initiated TPE in the middle phase of the clinical course, we were able to commence TPE within eight hours of admission. This early intervention may have played a pivotal role in preventing the progression of organ dysfunction and improving the patient’s outcome. This case is presented within the context of the existing literature on extracorporeal blood purification therapies for venom-induced organ dysfunction.

## Case presentation

Case presentation at the emergency department

A 78-year-old woman presented with a chief complaint of multiple hornet stings. At approximately 1:00 p.m. on the day of admission, she was stung by a swarm of hornets during a mountain hike and became immobile, prompting an emergency call. Upon the arrival of emergency medical services, she was in shock with impalpable radial pulses and was promptly transported to our emergency department. Her medical history included hypertension, diabetes mellitus, and dyslipidemia. She had no history of allergy or asthma and reported no history of smoking or alcohol consumption.

**Figure 1:**
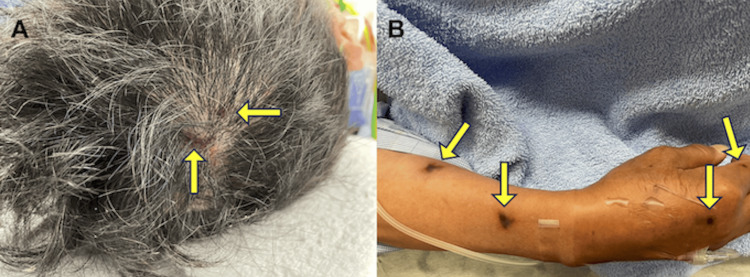
Sites of wasp stings (A, B) The image shows wasp stings at a total of 78 sting sites were identified: 29 on the scalp, one on the neck, one on the back, 22 on the left upper extremity, 12 on the right upper extremity, eight on the left lower extremity, and eight on the right lower extremity. Yellow arrows indicate the sting sites with retained wasp stingers.

Her height and weight were 145 cm and 58 kg, respectively. On arrival, her Glasgow Coma Scale (GCS) score was E3V4M6. Vital signs included a heart rate of 82 beats/min, blood pressure of 84/51 mmHg, respiratory rate of 28 breaths/min, and oxygen saturation of 100% while receiving 10 L/min of oxygen via a reservoir mask. Her airway was patent, without hoarseness, and her breath sounds were clear bilaterally, without stridor or wheezing. Radial artery pulses were faint, although peripheral cyanosis and facial pallor were absent. Widespread urticarial eruptions were observed across the body. A total of 78 sting sites were identified: 29 on the scalp, one on the neck, one on her back, 22 on the left upper extremity, 12 on the right upper extremity, eight on the left lower extremity, and eight on the right lower extremity (Figure 1). The yellow arrows in Figure 1 indicate the sting sites where the wasp stingers were lodged.

Initial blood tests revealed hepatic injury (total bilirubin (T-Bil), 0.9 mg/dL; aspartate aminotransferase (AST), 2,236 IU/L; alanine aminotransferase (ALT), 628 IU/L; alkaline phosphatase, 185 IU/L; lactate dehydrogenase (LDH), 1,143 U/L), renal dysfunction (blood urea nitrogen (BUN), 23.8 mg/dL; creatinine (Cr), 1.14 mg/dL), and elevated creatine kinase (CK), 194 U/L. Coagulation parameters showed a prothrombin time-international normalized ratio of 1.64 and an activated partial thromboplastin time >200 s (Table 1).

**Table 1:** Laboratory data at the emergency department Laboratory data were obtained on arrival in the emergency department (ED). Pre-TPE values were obtained immediately before initiation of TPE, and post-TPE values were obtained approximately 24 hours after completion of TPE. Abbreviations: TPE, therapeutic plasma exchange; CBC, complete blood count; TP, total protein; Alb, albumin; TB, total bilirubin; AST, aspartate aminotransferase; ALT, alanine aminotransferase; ALP, alkaline phosphatase; LDH, lactate dehydrogenase; Amy, amylase; BUN, blood urea nitrogen; Cr, creatinine; CK, creatine kinase; Na, sodium; K, potassium; Ca, calcium; Glu, glucose; CRP, C-reactive protein; WBC, white blood cell; RBC, red blood cell; Hb, hemoglobin; Hct, hematocrit; Plt, platelet; PT, prothrombin time; PT-INR, prothrombin time-international normalized ratio; APTT, activated partial thromboplastin time; BE, base excess; PCO₂, partial pressure of carbon dioxide; HCO₃⁻, bicarbonate

Biochemistry/Electrolyte	ED	Pre-TPE	Post-TPE	Normal range
AST (IU/L)	2,236	5,685	2,362	13-30
ALT (IU/L)	628	1,563	742	7.0-23
LDH (IU/L)	1,143	2,800	1,803	124-222
BUN (mg/dL)	23.8	23	24.6	8.0-20
Cr (mg/dL)	1.14	1.35	1.12	0.46-0.79
CK (IU/L)	194	787	19,815	41-153
Na (mEq/L)	143	142	145	138-145
K (mEq/L)	3.5	3.4	4.1	3.6-4.8
Ca (mg/dL)	9	8.8	8.9	8.0-10
CBC/Coagulopathy				
WBC (×10^3^/μL)	8.2	10.6	14.2	3.3-8.6
RBC (×10^4^/μL)	400	425	514	350-510
Hb (g/dL)	12.9	14	16.4	11.6-14.8
Hct (%)	38.1	41.2	48.5	35.1-44.4
Plt (×10^4^/μL)	22.5	22.5	23.3	15.8-34.8
PT (%)	48.3	60.5	62.9	70-130
PT-INR	1.64	1.39	1.35	-
APTT	>200	>200	115.5	23-38

Initial management

**Figure 2:**
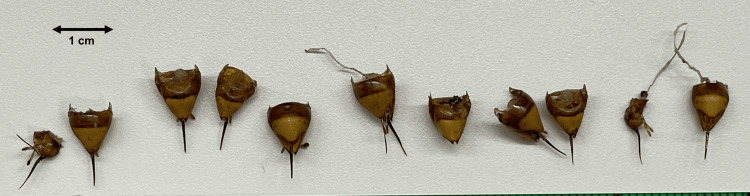
Embedded stingers with attached venom sacs Eleven stingers, each with an attached venom sac, remained embedded in the scalp. The stings were removed manually using forceps in the emergency department.

The patient was treated for anaphylactic shock with intramuscular adrenaline (0.3 mg), which resulted in rapid hemodynamic recovery (blood pressure: 123/64 mmHg) and improved consciousness (GCS: E4V5M6). Initially, the radial pulse was weak and barely palpable; however, it became clearly palpable after intramuscular adrenaline administration. Eleven stingers, including attached venom sacs, remained embedded in her scalp and were immediately removed manually using forceps (Figure 2).

The patient was administered D-chlorpheniramine maleate (5 mg), famotidine (20 mg), and methylprednisolone (125 mg). Reddish-brown urine was observed upon arrival, and laboratory findings and clinical features indicated MOF and disseminated intravascular coagulation (DIC). Because fibrin/fibrinogen degradation product (FDP) levels were not measured, the diagnosis of DIC was made clinically based on laboratory findings and clinical presentation. We discussed other possible causes of multi-organ dysfunction, including septic shock and other toxic or inflammatory processes; however, the temporal relationship with multiple hornet stings, the laboratory findings, and the absence of alternative etiologies supported the diagnosis of venom-induced systemic toxicity.

Management of DIC focused on treating the underlying causes, including anaphylaxis and venom-induced systemic toxicity, with supportive therapy. Currently, no specific antivenom or antidote is available for hornet envenomation; therefore, treatment relies primarily on supportive management. Her Wasp Sting Severity Score (WSS) - a clinical scoring system based on the number of stings, LDH, T-Bil, and the presence of tea-colored urine - was 16, prompting consideration of extracorporeal blood purification therapies. She was admitted to the intensive care unit (ICU) for close monitoring and therapeutic intervention.

ICU clinical course

**Figure 3:**
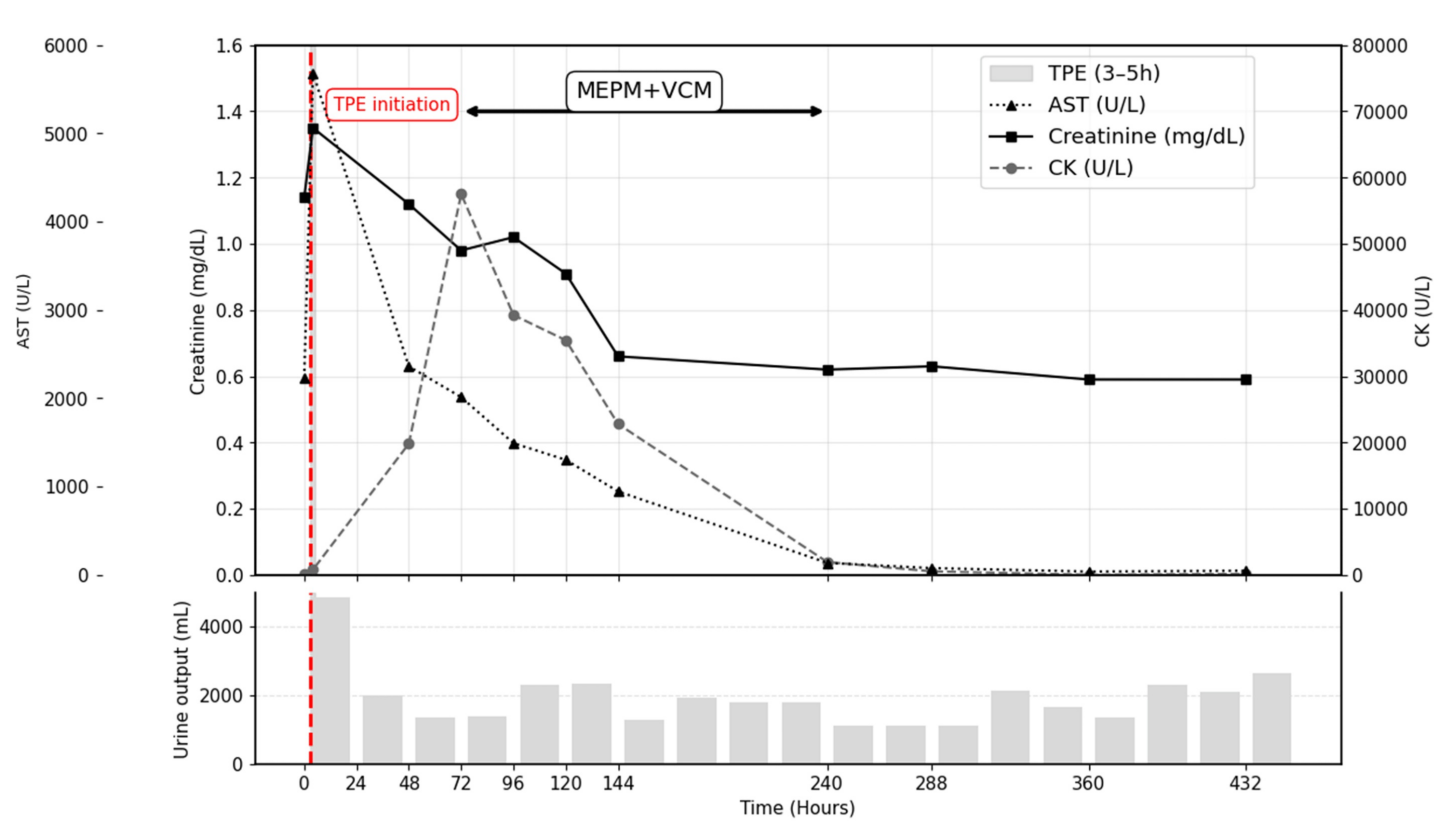
Time course of laboratory parameters following wasp envenomation and therapeutic interventions Trends of aspartate aminotransferase (AST), serum creatinine, and creatine kinase (CK) over time after admission are shown. A dashed vertical line indicates the initiation of therapeutic plasma exchange (TPE), which was performed eight hours after admission and lasted for 120 minutes (dark gray area). Empiric antibiotic therapy with meropenem and vancomycin (MEPM + VCM) was administered for seven days, starting 72 hours after admission (light gray area), to treat hospital-acquired pneumonia. AST and creatinine levels showed early improvement following TPE, whereas CK levels peaked around 96 hours and subsequently declined. All laboratory values were normalized by day 10, with no rebound observed thereafter.

After four hours of admission, laboratory tests indicated worsening hepatic function (AST, 5,685 IU/L; ALT, 1,563 IU/L) and renal function (BUN, 23.0 mg/dL; Cr, 1.35 mg/dL). A central venous catheter was inserted, and TPE was initiated eight hours after admission. Plasma separation was performed using an OP-08D plasma filter (Plasmaflo®, Asahi Kasei Medical Co., Tokyo, Japan), with a plasma exchange-selective plasma filtration and dialysis circuit employed. Plasma volume was estimated at 2.05 L using the formula: $45~\mathrm{kg} \times 13 \times \frac{1 - \mathrm{hematocrit}}{100}$. A total of 20 units of fresh-frozen plasma - derived from human blood and defined as approximately 120 mL per unit under the Japanese standard (total volume approximately 2.4 L) - were exchanged over 120 minutes. The patient’s clinical course is shown in Figure 3. Throughout this period, the patient remained fully conscious, with a GCS score of E4V5M6.

The patient developed progressive hypoxemia, requiring oxygen escalation to a non-rebreather mask at 9 L/min. Before TPE, laboratory findings showed worsening organ dysfunction, with elevated AST (5,685 U/L; normal: 13-30 U/L), ALT (1,563 U/L; normal: 7-23 U/L), CK (787 U/L; normal: 41-153 U/L), and serum Cr (1.35 mg/dL; normal: 0.46-0.79 mg/dL). Following TPE (day 2), marked improvements were observed in hepatic and renal parameters: AST decreased to 2,362 U/L, ALT to 742 U/L, and serum Cr to 1.12 mg/dL. In contrast, CK levels increased markedly to 19,815 U/L, indicating ongoing muscle injury despite improvement in other organ systems (Table 1).

On day 2, oxygen requirements decreased to 2 L/min via nasal cannula. On day 3, hoarseness and inspiratory stridor were observed. At the time of respiratory deterioration, laryngoscopy revealed swelling of the left arytenoid region, but no evidence of critical airway obstruction. Because the patient’s respiratory status stabilized with non-invasive positive pressure ventilation (NIPPV), endotracheal intubation was deferred, and the patient was closely monitored for potential airway compromise. Chest radiography demonstrated a unilateral infiltrate in the right lower lobe. These findings were not consistent with transfusion-related acute lung injury (TRALI), which typically presents with bilateral pulmonary infiltrates, and the respiratory deterioration was therefore considered more consistent with pneumonia. Meropenem plus vancomycin was initiated for presumed nosocomial pneumonia.

Systemic corticosteroids were administered only at presentation and were not continued beyond the initial management. Supportive care included careful intravenous fluid administration and close hemodynamic monitoring. Renal function was monitored using both serum Cr levels and urine output. The patient consistently maintained urine output exceeding 1 mL/kg/h, and serum Cr levels showed a trend toward improvement following TPE. Therefore, renal replacement therapy was considered but ultimately not required.

On day 4, CK levels peaked at 39,239 U/L, and the patient was weaned off NIPPV the same day. She was transferred from the ICU to a general ward on day 5. Antibiotics were discontinued on day 10. By day 10, laboratory parameters showed marked improvement, with AST 136 U/L, ALT 415 U/L, CK 1,938 U/L, and serum Cr 0.62 mg/dL. The patient made a favorable recovery, being discharged home on day 22. Since discharge, the patient has been followed annually in the outpatient clinic. Routine laboratory testing, including hepatic and renal function tests, has not been performed during follow-up; therefore, objective confirmation of post-discharge hepatic and renal function is unavailable. We summarized the clinical course as a brief tabular timeline (Table 2).

**Table 2:** Clinical timeline of symptom progression and therapeutic interventions

Time point	Clinical events and findings
Day 0 (injury and admission)	The patient sustained multiple hornet stings during a mountain hike and was found in shock by emergency medical services. On arrival at the emergency department, she was hypotensive with impaired consciousness. Initial laboratory tests revealed acute hepatic injury, coagulopathy, and early renal dysfunction. She was diagnosed with anaphylactic shock with venom-induced systemic involvement and admitted to the intensive care unit (ICU) on the day of presentation.
Within 8 hours after admission	Despite initial hemodynamic stabilization, laboratory parameters demonstrated rapid progression of organ dysfunction. Therapeutic plasma exchange (TPE) was initiated approximately eight hours after hospital admission during the early phase of multiple organ failure.
Day 2	After TPE, hepatic and renal function showed improvement, as evidenced by decreasing aminotransferase levels and improving serum creatinine. Renal function was assessed using both serum creatinine and urine output; the patient consistently maintained urine output greater than 1 mL/kg/h. In contrast, creatine kinase levels increased markedly, indicating ongoing rhabdomyolysis.
Days 3-4	The patient developed transient respiratory deterioration requiring non-invasive positive pressure ventilation. Liver and kidney function continued to improve. Creatine kinase levels peaked on day 4 and subsequently began to decline.
Day 5	Respiratory status stabilized, and the patient was transferred from the ICU to a general ward.
Day 10	Laboratory parameters, including hepatic enzymes, renal function, and creatine kinase levels, returned to within normal ranges without evidence of rebound deterioration.

## Discussion

This case highlights the successful use of early extracorporeal blood purification in the management of venom-induced MOF resulting from wasp envenomation. The initial hypotension was attributed to anaphylactic shock and was promptly treated with intramuscular adrenaline. However, subsequent laboratory findings revealed evolving MOF and DIC, prompting a rapid transition to intensive care management. Although no standardized treatment protocol exists for severe cases of wasp venom toxicity, the decision to initiate TPE in this case was based on worsening organ dysfunction observed during follow-up laboratory assessments [8]. TPE is an extracorporeal blood purification technique in which a patient’s plasma is removed and replaced with other replacement fluids, in order to eliminate circulating toxins, inflammatory mediators, or pathogenic substances. In the following section, we focus on the rationale for selecting TPE, and current considerations regarding the indications for extracorporeal blood purification in severe cases of wasp envenomation.

There is a lack of robust evidence regarding the efficacy of extracorporeal blood purification therapies for wasp venom toxicity. In particular, debate persists over the optimal modality - plasma exchange versus hemodialysis - each with distinct theoretical advantages and limitations [8,9]. First, the choice of modality should consider the molecular characteristics of wasp venom components, which can be broadly classified into three categories: amines (such as serotonin and histamine), peptides (such as mastoparan and kinins), and enzymes (such as phospholipase A1 and hyaluronidase) [3,4]. Among these, enzymes are thought to exert the most potent cytotoxic effects. The molecular weight of wasp venom constituents is generally <140 kDa, with cytotoxic enzymes primarily ranging between 25,000 and 45,000 Da [3,4,10]. Hemodialysis is effective for molecules <approximately 2,000 Da, making it unsuitable for removing higher-molecular-weight toxins. Therefore, if the primary therapeutic goal is to reduce circulating venom levels, plasma exchange should be considered the most appropriate modality. It is important to note, however, that animal studies using rat models have shown that venom components are rapidly cleared from the bloodstream within several hours [11], raising questions regarding the temporal window and utility of blood purification therapies targeting the venom itself. Consequently, attention has shifted toward addressing downstream inflammatory responses. Wasp venom-induced MOF is believed to involve a complex interplay of inflammatory mediators, including interleukins and interferon gamma [12], providing further justification for selecting a modality capable of removing such mediators. Some experts advocate for blood purification techniques aimed at eliminating larger molecules, such as hemofiltration or TPE [5]. Case reports have also described the use of direct hemoperfusion with polymyxin B-immobilized fiber columns (PMX-DHP) and continuous hemodiafiltration using AN69ST hemofilters as potentially effective options [13]. Other experts recommended combining TPE with continuous renal replacement therapies, such as continuous hemofiltration or hemodiafiltration [9]. As discussed above, we selected TPE as the primary modality. 

Regarding the optimal number of TPE sessions, existing literature and expert opinions offer limited guidance. Therefore, clinicians should tailor the number of sessions to each case, considering the target molecules and the patient’s clinical course. In our case, TPE was primarily intended to eliminate circulating venom. Our literature review revealed no reports suggesting persistent or rebound elevations in venom levels after a single session. We, therefore, considered that one session of TPE was sufficient for venom removal. Furthermore, the patient’s favorable clinical course, including adequate urine output and improvement in laboratory data, supported our decision not to perform additional extracorporeal therapies.

The criteria for initiating blood purification remain undefined; however, several studies have proposed using the number of sting sites (>50) as a threshold [5,6], while others have suggested scoring systems such as the WSS, which incorporates four variables (presence of brown urine, number of stings, LDH, and T-Bil) [14], with a cutoff score of ≥3 as a marker for identifying candidates for TPE. In our case, the WSS was 16, clearly exceeding this proposed threshold. Previous studies have also suggested that early initiation of blood purification therapy may be associated with improved mortality outcomes [14]. In the present case, TPE was initiated within eight hours of admission, which may have contributed to the favorable clinical course. Further investigation is warranted to determine the optimal timing for early intervention and to establish standardized criteria for blood purification therapies.

## Conclusions

Early initiation of intensive care is essential in cases of venom-induced MOF, particularly when accompanied by extensive stings and DIC. In this case, early TPE was associated with rapid improvement in hepatic and renal dysfunction, with AST decreasing from 5,685 U/L to 2,362 U/L, and serum Cr improving from 1.35 mg/dL to 1.12 mg/dL after a single session. This case suggests that early plasma exchange may play a beneficial role in selected cases of venom-induced MOF when initiated promptly alongside supportive therapy. As this is a single case, these findings should be interpreted with caution, but they underscore the potential value of early TPE in similar clinical scenarios. Further studies are warranted to clarify the efficacy, optimal modality, and timing of blood purification therapy in wasp venom toxicity.
